# Changes in prevalence of cognitive impairment and associated risk factors 2000–2015 in São Paulo, Brazil

**DOI:** 10.1186/s12877-021-02542-x

**Published:** 2021-10-27

**Authors:** Fabiana Silva Ribeiro, Yeda Aparecida de Oliveira Duarte, Jair Lício Ferreira Santos, Anja K. Leist

**Affiliations:** 1 Department of Social Sciences, University of Luxembourg, Esch-sur-Alzette, Luxembourg; 2 School of Public Health, University of São Paulo – São Paulo (SP), São Paulo, Brazil; 3 Department of Social Medicine, University of São Paulo – Ribeirão Preto (SP), Ribeirão Preto, Brazil

**Keywords:** Cognitive impairment, prevalence, risk factors, protective factors, ageing

## Abstract

**Introduction:**

Decreases in prevalence of cognitive impairment and dementia over the last two decades have been observed in different countries for cohorts entering older age. This study aimed to assess the cognitive impairment prevalence and explore associated factors among subjects aged >60 living in São Paulo, Brazil.

**Method:**

Data came from a population-based Health, Welfare and Aging survey conducted in 2000, 2006, 2010, and 2015. Cognitive impairment was detected using the abbreviated Mini-Mental State Exam corrected by formal education years. In total, there were 5922 respondents in the statistical analyses.

**Results:**

Logistic regression models adjusted for age group, income, race, cardiovascular risk factors, and depression were used to estimate cognitive impairment prevalence. Between 2015 and 2000, respondents were more likely to report formal education, hypertension, diabetes, and overweight/obesity. Moreover, the weighted analyses showed that cognitive impairment prevalence was higher in 2015, even adjusting for sociodemographic and socioeconomic characteristics, cardiovascular risk factors, and depression.

**Conclusion:**

In contrast to decreases in cognitive impairment prevalence in other countries and despite increases in educational years, our findings suggest no secular improvements in cognitive health for the 2015 wave of older adults residing in São Paulo.

**Supplementary Information:**

The online version contains supplementary material available at 10.1186/s12877-021-02542-x.

## Background

The Brazilian population of 60 years old or older grew by about 4.8 million between 2012 and 2017, bringing it to a total of more than 30.2 million in 2017 [1]. A large share of older people live in the most populated cities, as is the case of São Paulo, which, according to the Brazilian Institute of Geography and Statistics [2], comprised 15.71% of the older population in 2018.

Furthermore, Brazil is expected to become the sixth oldest country in the world by 2025 [3]. As a consequence of the rise in numbers of older adults, age-related diseases, such as cognitive impairment, are expected to increase in prevalence resulting from different diseases or/and injuries that affect the brain [4].

Interestingly, most of the recent studies from high-income countries have shown a decrease in the prevalence of cognitive impairment and/or dementia over the last two decades for more recently born cohorts entering older age [5–9]. However, the opposite pattern was observed in two systematic reviews evaluating the prevalence of cognitive impairment and dementia in low-and middle-income countries of Latin America and the Caribbean. The authors showed that more recently published studies showed a higher prevalence, both associated with higher age, being women, lower educational levels, and living in rural regions [10, 11].

Congruently with the above finding, observing the few studies published in the field over the years in Brazil, it is possible to notice a higher prevalence of cognitive impairment for adults over 60 years of age in more recent studies. For instance, the prevalence rate in samples collected until 2000 was between 11.5 and 19.2% [12–15], while the studies conducted in 2008 [16] and 2011 [17] showed a prevalence of 34.1 and 38.9%, respectively. For this reason, it is critical to identify the trends in cognitive impairment of representative samples across the years to explore the main protective and risk factors to its development, and as a result to provide effective training for health professionals, strategically allocate investments of governments [18], and develop populational health policies [19].

According to the previous literature, higher educational levels were consistently associated with lower likelihood of developing cognitive impairment or dementia. These results are explained by the capability of education to promote cognitive reserve, which is assumed to increase adaptability of the brain to compensate neuropathological and vascular damage before showing cognitive impairment or/and dementia symptoms [20]. Furthermore, higher educational levels seem to be related to healthy behaviours, better access to health, better-paid professions, and higher cognitive functioning [6, 21].

On the other hand, higher age seems to be a risk factor for cognitive impairment development. Further, samples from a less privileged socioeconomic background seem to develop cognitive impairment earlier than samples drawn in countries with higher economic development [22, 23]. Moreover, some studies worldwide have observed a higher prevalence of cognitive impairment for blacks than whites [16, 24, 25]. Thus, differences in education, health, socioeconomic background, cultural factors [26, 27], and structural discrimination might explain a higher prevalence of dementia for blacks [28].

Furthermore, less privileged socioeconomic background could be associated with lower educational attainment [6, 13, 14, 22, 23, 26], early onset of vascular risk factors [23], systemic, or/and metabolic disorders, as well as mood disorders, such as depression [16, 22, 29], which is frequently not included in studies exploring the cognitive impairment prevalence [30].

Although preliminary evidence from high-income countries suggests that there has been a reduction in the rate of older adults with cognitive impairment, as far as we know, this trend has not been investigated in Brazil yet. Furthermore, another critical motivation to carry out this study was the negative and positive changes observed in the country since the beginning of the SABE study. During the time this study was conducted, 2000–2015, Brazil was experiencing an increase in the prevalence of overweight people, obesity, diabetes, systemic arterial hypertension, and cardiovascular diseases [31, 32]. On the other hand, Brazil experienced decreases in socioeconomic inequalities with income rises that lasted until 2014 [33], as well as an increase in educational background of the population [34] and improvements in access to public health care [35].

In this context, the goal of our study was to evaluate the recent trends in cognitive impairment across four waves of the Health, Well-being and Aging survey (SABE) with data collected in representative samples of São Paulo, Brazil, in 2000, 2006, 2010, and 2015, as well as estimate age-specific prevalence. Besides, according to previous literature, we also sought to explore the risk and protective factors associated with cognitive impairment. We hypothesise that due to the improvement of socioeconomic factors, the São Paulo population entering older age in more recent waves would show higher educational levels and income, leading to a lower prevalence of cognitive impairment.

## Methods

### Data and Sample

This study used data from four waves of SABE study (Health, Well-Being, and Aging) 2000, 2006, 2010, and 2015 including a representative sample of older adults above 60 years old living in the state of São Paulo. The SABE study has been following respondents longitudinally with assessments every five years, however, in order to maintain population representativeness, new cohorts were included at the different waves of SABE study. In this study, our analyses represent a time-series trend using waves as cross-sectional representative samples. For this reason, participants who responded in any wave of the study were included in the statistical analyses. Thus, participants interviewed in more than one survey were included in our statistical analyses for every wave to which they contributed, and specific weights were applied for each wave. We followed the methodology used by Langa et al. [6].

The SABE study interviews were face-to-face and household-based, conducted by trained interviewers in Portuguese, the official language of Brazil. Moreover, all participants answered the same order of questions with the same wording. For further specifications of the sampling design, such as the geographic areas of São Paulo city being included in two-stage stratified sampling method, see Lebrão et al. [36]. The sample composition of each wave of the SABE study is provided in Table 1.

**Table 1:** Composition of the four waves samples of the SABE study

Waves			
2000	2006 (***n*** = 1413)	2010 (***n*** = 1346)	2015 (***n*** = 1224)
(*n* = 2143)	Re-assessed (*n* = 1115)	Re-assessed (*n* = 990) (Wave 2000 = 748) (Wave 2006 = 242)	Re-assessed (*n* = 838) (Wave 2000 = 382) (Wave 2006 = 186) (Wave 2010 = 270)
	Deaths (*n* = 491, 22.9%)	Deaths (*n* = 169, 11.9%)	Deaths (*n* = 308, 22.9%)
	Refusal/not located or institutionalized (*n* = 537, 25.1%)	Refusal/not located or institutionalized (*n* = 254, 18%)	Refusal/not located or institutionalized (*n* = 200, 14.8%)
	New participants (*n* = 298)	New participants (*n* = 356)	New participants (*n* = 386)

Participants took part in research voluntarily, and all of them signed an informed consent form. The Human Research Ethics Committee at the School of Public Health, University of São Paulo, and the National Committee for Research Ethics approved the SABE study. Furthermore, the ethical standards of the Cognitive Aging research project that this study is part of were approved by the Ethics Review Committee of the European Research Council in November 2018.

In this study, we excluded those participants without cognitive assessment, moreover, as we had missing data for some of the risk factors included, i.e., cardiovascular and/or depression, we performed imputation, which is a statistical technique for dealing with missing data. Then, we conducted sensitivity analyses with the aim to observe whether statistical results would be different between the imputed and non-imputed datasets, and it was observed that both datasets led to similar outcomes. For this reason, in this study, we report the statistical analysis using original data without imputation.

### Measures

#### Cognitive functions - Dependent variable

The cognitive screening was carried out with the abbreviated version of the Mini-Mental State Examination (MMSE) validated by the World Health Organization with a low-educated population, and as a result, less dependent on the possible effects of schooling [37, 38]. This MMSE version comprises 13 items with a maximum score of 19 points and originally included a cut-off point of <= 12 points indicating cognitive impairment. However, as shown in Andrade et al. [39] using SABE data, the MMSE might be sensitive to differences in education. For this reason, the cut-off scores for cognitive impairment were based on the 10th percentile of the MMSE score for each education category, thus we applied an education-specific cut-off point as follows: MMSE score < = 9 for no years of formal schooling, MMSE score < = 12 for 1 or 2 years of schooling, MMSE score of <= 14 for 3 to 8 years of education, and MMSE score of <= 16 for those with nine or more years of schooling. Participants responded to the MMSE without the help of informants, in the case of those who were unable to respond to the MMSE, a score of zero was given. In this sense, we included any cognitive impairment in our analysis, which means cognitive impairment without and with dementia.

#### Measures used as covariates

Sociodemographic and socioeconomic characteristics, risk factors commonly known to increase cardiovascular risk [40], and depression were used as covariates. Sociodemographic characteristics included sex (male/female), age in age brackets (60–64, 65–69, 70–74, 75–79, 80–84, and ≥ 85 years), race (White, brown or mixed, black, and other) and individual income as the number of times the national minimum wage (< 1, 1–2, 2–3, 3–4, and > 4). Cardiovascular risk factors comprised self-reported heart disease, stroke, diabetes, hypertension, and BMI (<18.5, 18.5–24.9, 25–29.9, 30+). In the following, we provide more detail for some of the covariates.

Race categories were classified according to the participants’ self-response to a list of possible answers corresponding to the official classification of self-reported skin colour in Brazil [41]; white, brown or mixed, black, yellow, and indigenous. In this study, we included respondents self-classifying as yellow or indigenous as ‘other’ due to low case numbers for these categories.

The individual income was calculated according to the national minimum wage (NMW) of the year of the interview, which was as follows: 2000 (R$151), 2006 (R$350), 2010 (R$510), 2015/2016 (R$788-R$880). The wage in R$ was recalculated by how many times the NMW would be multiplied to reach the same amount (1–2 times to >4 times).

Self-reported cardiovascular risk factors were assessed by the following questions for each of the variables (heart disease, i.e., congestive heart failure, coronary heart disease or occurrence of a heart attack, stroke, diabetes, hypertension): “Has a doctor or nurse ever told you that you had....?”

Body weight was objectively measured using a calibrated scale and height by applying a stadiometer. Body mass index (BMI) was calculated by participants’ weight in kilograms (W) and height in meters (h), and we applied the following formula: *BMI = W/h*^*2*^*.*

Depressive symptoms were assessed using the abbreviated Geriatric Depression Scale [42], which is one of the most used scales to detect severe and mild depressive symptoms in older adults and has been used in previous publications of SABE project [43, 44]. The number of depressive symptoms was used to categorise no, mild, and severe depression. The cut-off for mild depression goes from 6 to 10 points and for severe depression is 11 points or more.

Finally, following consistent literature in the field, we included prior test exposure as a dichotomous variable, indicating whether or not a participant had been previously tested to adjust for possible effects of repeat testing in this sample [6, 45, 46].

#### Statistical Analysis

The cross-sectional sample weights were presented for each wave of the SABE study according to population census. Therefore, the weighted waves of data derive population-representative estimates for the population of older adults of São Paulo city in the years 2000, 2005, 2010, and 2015. Firstly, we applied descriptive analyses to show percentage distributions for sociodemographic and socioeconomic characteristics, cardiovascular risk factors, and depression at each wave. Then, to observe possible differences among the four waves in cognitive impairment, age, sex, race, education, income, cardiovascular disease, BMI, and depression levels, we carried out the Rao Scott chi-square test, which accounts for the complex survey design. Supplementary Table S1 displays the descriptive analysis for all four waves.

Secondly, to assess the temporal trend in the prevalence of cognitive impairment and which independent variables would influence it across the four years, we ran logistic regression models, in which cognitive impairment was used as a dependent dichotomous variable. The reference group was the one with intact cognitive performance.

We separately performed four logistic regression models including different sets of independent variables added consecutively, as follows; Model 1 included wave year only; Model 2 additionally included age groups; Model 3 comprised sociodemographic covariates, such as education, race, and wage; and finally, Model 4 adjusted for age group, educational attainment, self-reported heart disease, stroke, diabetes, hypertension, BMI, and depression levels to explore changes in the prevalence of cognitive impairment across waves after adjustment of these factors. In addition, we included in the analyses the sampling weights to adjust the complex sampling design of the SABE survey and an indicator of first testing for the panel sample to adjust for the effect of repeated assessment.

Moreover, to adjust for repeated samples across waves, we used the STATA (release 16, Stata Corp) *svyset* command in analyses setting for complex data, in which respondent identity was defined as a cluster variable. Therefore, the statistics were weighted to account for oversampling and poststratification adjustments to each population year survey estimates, and standard errors were adjusted at the level of the five geographical areas (i.e., of São Paulo city regions.

STATA (release 16, Stata Corp) software was used to conduct all statistical analyses.

## Results

The descriptive analysis of sociodemographic and socioeconomic characteristics, and prevalence of cardiovascular risk factors and depression showed that, compared with the 2000 cohort, the 2015 cohort was more likely to report some formal education (91.3% in 2015 vs. 80% in 2000). Moreover, we observed that respondents were more likely to receive equivalent to 1 to 2 NMW in 2015–49% (95% CI, 45.0–53.5%) compared to 2000–16.8% (95% CI, 14.5–19.5%).

Regarding cardiovascular risk factors, in 2015 compared to 2000, respondents were increasingly more likely to report diabetes, 28.3% (95% CI, 25.4–31.2%) vs. 18.5% (95% CI, 16.4–20.8%), hypertension, 66.3% (95% CI, 63.2–69.3%) vs. 53.3% (95% CI, 50.4–56.3%), be overweight/obese, 32.8% (95% CI, 29.0–36.8%), vs. 22.1% (95% CI, 20.1–24.3%) (*p* < .001), no differences in prevalence of mild or severe depression were found across waves.

Moreover, females compared to males were more likely to report hypertension, 61.7% (95% CI, 59.0–64.4%) vs. 38.3% (95% CI, 35.6–41.0%), be overweight/obese, 35.2% (95% CI, 32.1–38.4%), vs. 18.8% (95% CI, 15.9–22.0%), and present mild, 17.1% (95% CI, 15.4–18.9%) vs. 9.9% (95% CI, 8.4–11.6%), or severe depression symptoms, 4.7% (95% CI, 3.8–5.8%) vs. 2.0% (95% CI, 1.3–2.9%) (all *ps* < .001). For a more detailed description of the sample, see Table S1 provided in the supplementary material.

**Fig. 1:**
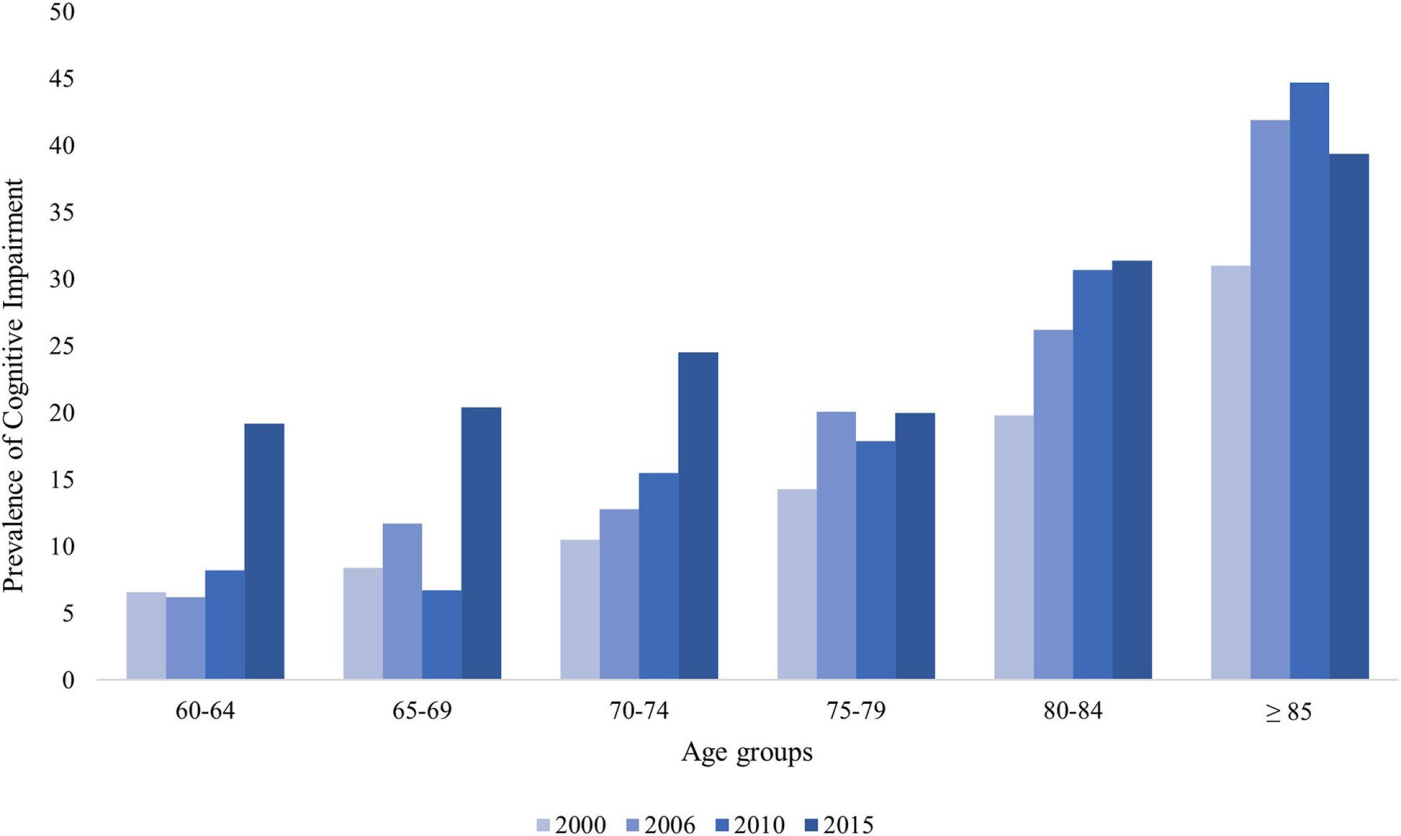
Prevalence of cognitive impairment by age groups

As displayed in Fig. 1, our analysis regarding the proportion of cognitive impairment across waves showed that prevalence was higher in 2015 (23.2% [95% CI- 20.2 - 26.4%]) compared to 2000 (10.2% [95% CI- 9.0 - 11.6%]), 2006 (13.9% [95% CI- 11.8 – 16.3%]), and 2010 (14.8% [95% CI- 12.6 – 17.4%]). Specifically, higher cognitive impairment prevalence was observed for those participants between 60 and 79 years old (For more information, see supplementary material, Table S1).

In Table 2, we report the results Odds Ratio (OR) and 95% confidence interval for the four regression models. In the unadjusted Model 1, a significantly higher prevalence of cognitive impairment was observed in 2006, 2010, and 2015 compared to 2000. Even after controlling for age brackets, this higher prevalence in later survey years remained (Model 2), however adjusting for sociodemographic and socioeconomic characteristics (Model 3), only a higher prevalence of cognitive impairment for 2015 compared to 2000 persisted. Aiming to understand whether any of the covariates could explain the prevalence of cognitive impairment between 2000 and 2015, we run the model 3, including only one additional covariate at a time, and none of the variables determined the differences in prevalence in cognitive impairment between 2000 and 2015.

**Table 2:** Odds Ratios of prevalence of cognitive impairment across the four waves

Variables	Models- OR (95% CI)			
	1 (*n* = 5922)	2 (*n* = 5922)	3 (*n* = 5154)	4 (*n* = 4409)
2000	(reference)	(reference)	(reference)	(reference)
2006	1.41 (1.11–1.81)**	1.34 (1.04–1.71) **	1.35 (0.84–2.19)	1.39 (0.80–2.42)
2010	1.53 (1.22–1.92) **	1.38 (1.10–1.72) **	1.41 (0.91–2.22)	1.52 (0.91–2. 54)
2015	2.65 (2.13–3.29) **	2.40 (1.89–3.04) **	2.39 (1.43–4.01) **	3.03 (1.71–5.36) **
Age-y^a^				
60–64		(reference)	(reference)	(reference)
65–69		1.20 (0.90–1.59) **	1.26 (0.78–2.02)	1.36 (0.81–2.30)
70–74		1.69 (1.23–2.31) **	1.78 (1.06–2.99)*	1.95 (1.07–3.58)*
75–79		1.90 (1.41–2.56) **	2.09 (1.27–3.44)*	1.89 (1.07–3.36)*
80–84		3.40 (2.49–4.65) **	3.80 (2.25–6.40) **	3.13 (1.67–5.88) **
≥ 85		5.41 (4.07–7.20) **	5.22 (3.05–8.93) **	4.08 (2.15–7.74) **
Sex^b^				
Female			(reference)	(reference)
Male			1.21 (.98–1.49)	1.21 (0.96–1.53)
Race^b^				
White			(reference)	(reference)
Mixed			1.27 (0.98–1.49)	1.24 (0.93–1.67)
Black			1.68 (1.16–2.43)**	1.66 (1.11–2.46)*
Other			1.09 (0.73–1.61)	1.17 (0.74–1.83)
Number of wages				
< 1			(reference)	(reference)
1–2			0.70 (0.52–0.93)*	0.73 (0.54–0.99)*
2–3			0.65 (0.46–0.90) *	0.74 (0.52–1.06)
3–4			0.65 (0.45–0.93) *	0.78 (0.52–1.16)
> 4			0.53 (0.35–0.79) **	0.68 (0.44–1.05)
Cardiovascular risk factors				
No Stroke				(reference)
Stroke				1.92 (1.34–2.74)**
No diabetes				(reference)
Diabetes				1.09 (0.82–1.45)
No heart disease				(reference)
Heart disease				0.91 (0.70–1.20)
No hypertension				(reference)
Hypertension				1.27 (0.95–1.69)
BMI				
< 18.5				(reference)
18.5–24.9				1.51 (0.68–3.36)
25–29.9				1.61 (0.71–3.68)
≥ 30				1.13 (0.49–2.61)
Depression				
No depression				(reference)
Mild depression				1.87 (1.37–2.57)**
Severe depression				2.13 (1.23–3.68)**

Note: * *p* < 01, ** *p* < 001. *OR* odds ratio, *CI* confidence interval, *NMW* year-specific national minimum wage

After controlling for cardiovascular risk factors and depression (Model 4), higher likelihood of cognitive impairment was still observed in 2015 compared to 2000. Furthermore, only the history of stroke and the presence of depression contributed to explaining the prevalence of cognitive impairment. Moreover, white and younger respondents had a lower risk of cognitive impairment.

## Discussion

In this study, we sought to analyse the prevalence of cognitive impairment over 15 years (2000 to 2015) in older residents of São Paulo state, Brazil, and the possible factors contributing to cognitive impairment development. Recent studies from high-income countries suggest a stable or decreasing prevalence of cognitive impairment and dementia in more recently born cohorts [6, 47, 48]. In contrast, our Brazilian SABE data findings suggest a higher proportion of participants with cognitive impairment in 2015 than 2000, which seems congruent to recent systematic reviews exploring the topic in Latin America and the Caribbean [10, 11], and studies in Brazil [16, 17].

Regarding the risk factors associated with cognitive impairment, as expected, we found that older participants were more likely to present cognitive impairment that can be justified by the increase of chronic diseases with older age [49] or factors damaging the brain, such as head trauma [50]. This is congruent with the positive association observed in this study between cognitive impairment and history of stroke.

Moreover, we also observed that self-identifying as black was associated with significantly higher odds of cognitive impairment, which is in line with various studies carried out in Brazil [16], and in other countries such as the United States [23, 25], and the UK [24]. These findings might reflect two main factors: Firstly, minority populations are more likely to experience structural discrimination and childhood adversity, such as malnutrition, conflict, and family segregation. Secondly, the black Brazilian population has lower access to quality education than white people. Specifically, the black population is more likely to attend schools characterised by poor structured schedules and resources, less rigorous curricula, fewer classes offered, and teachers who  expect less from black than white students [51]. All these factors are linked to a lower potential to build up cognitive reserve during schooling, which may have resulted in the higher cognitive impairment rates of black respondents.

Furthermore, as expected, higher income was a protective factor to unimpaired cognitive functioning. Thus, income may be a general indicator of more advantaged socioeconomic conditions but may also, to some extent, reflect higher occupational complexity, which has been hypothesised to help mitigate the negative effects of brain changes in older age [52, 53].

Nevertheless, even with higher formal education and income, and additionally using education-specific cut-off scores of cognitive impairment, in line with Andrade et al. [39], we detected an elevated prevalence of cognitive impairment in 2015 compared with 2000. Downer et al. [54] pointed that longer schooling does not necessarily mean improvement in educational quality. It is crucial to consider this aspect since the ability to read and interpret are important learning outcomes with strong explanatory ability for cognitive function in older ages [55, 56].

Besides, it is essential to mention that although literacy of older adults in Brazil increased in the last 30 years, from 52 to 77%, the average years of education still rest at five years [57, 58], which is low compared to high-income countries [58]. In our sample, 92% of the respondents were likely to present more formal education in 2015, in contrast with 80% in 2000, however, around 60% of the sample had only primary school education. Out of the respondents in the U.S. Health and Retirement Study analysed by Langa et al. [6] a total of 79.6% of the respondents had at least 12 years of formal education, whereas only 30.2% of the respondents of SABE study presented here reported this level of education. With a generally low-educated sample like in the SABE study, primary school education may not be sufficient to promote cognitive reserve to prevent cognitive impairment.

Another risk factor for cognitive impairment was the existence of mild or severe symptoms of depression, cited as a consequence of lack of close social contacts [59], or stressful events during life, such as the death of a loved one, moving from work into retirement, or developing an illness [60, 61]. However, as the associations observed here are cross-sectional, the presence of depression could also be an early symptom, rather than a risk factor, of dementia [62].

Finally, although we have not observed associations between cognitive impairment and diabetes, heart diseases, hypertension, or BMI, we detected a higher prevalence of cardiovascular risk factors, specifically, overweight/obesity, as well as diabetes and hypertension in recent waves that possibly contributed to a higher cognitive burden. In fact, these results are congruent with changes in the nutrition of Brazilian older adults [63, 64]. Specifically, this trend is characterised by increases in the consumption of ultra-processed foods rich in sugar and fat [63]. Therefore, this result shows the necessity of public health education and programs regarding the importance of a healthy weight and leading a healthy lifestyle.

### Strengths and limitations

As far as we know, this is the first study analysing cognitive impairment across 15 years in the Brazilian older population. The prevalence of cognitive impairment in the other states of Brazil may be different, since São Paulo is the biggest city in Brazil, which attracts an influx of citizens with higher levels of education, higher diversity among its population, as well as characterised by comparatively higher inequalities [65].

The research design of the SABE allows us to provide estimates of cognitive impairment for different age groups over time, but we cannot exclude the possibility of bias due to selective enrolment. Moreover, the panel respondents could have benefited from repeated assessment leading to seemingly lower prevalence of cognitive impairment; however, as we adjusted to repeated testing and the waves were quite far apart, these concerns are alleviated.

We were able to investigate a large set of potential risk factors for cognitive impairment. Comparing assessments in 2000 and 2015, we observed increases in prevalence of both risk factors for cardiovascular disease and cognitive impairment. Regarding prevalence of risk factors for cardiovascular diseases, we cannot exclude the possibility that the numbers of undiagnosed participants could have decreased across time (from 2000 to 2015) due to improvement in access and use of healthcare and the increase in prevalence of cardiovascular conditions found could be the result of improvements in diagnosis. However, information on BMI, which also increased over time, is less subject to these limitations and related to cardiovascular risk factors [66, 67]. Furthermore, increases in prevalence of cardiovascular risk observed in this study are in line with findings from the Global Burden of Disease study [68]. However, other factors associated with increased risk of cognitive impairment, such as air pollution [69] that probably also increased across the observation window, were not assessed in this study.

It is important to clarify that in this study, we evaluate the prevalence of cognitive impairment, which is characterised by mnesic problems, difficulties in acquiring new knowledge, concentrating and/or make decisions, affecting typical daily routines, without differentiating subjects with mild cognitive impairment [70] or dementia [71]. Consequently, our cognitive impairment measure was quite crude to respect the limits of a comprehensive interview study as the SABE. Furthermore, we used a modified version of MMSE, which has some limitations, i.e., impossibility to make a differential diagnosis and also the use of different scores [72].

Future studies could use more extensive neuropsychological assessments to allow differential diagnoses, such as the different types of Mild Cognitive Impairment [70] and dementia.

## Conclusion

Our results highlight that São Paulo residents have not benefited from the secular improvements in cognitive health over the last decades observed in other countries. The findings point to the importance of intervention programs to promote cognitive reserve and a healthy lifestyle in the population at early old ages to prevent and reduce the risk of dementia.

## Supplementary Information

**Additional file 1: Table S1.** Characteristics of the four waves (weighted estimates).

## Data Availability

The datasets generated and/or analysed during the current study are not publicly available due to privacy and ethical restrictions but are available from the corresponding author on reasonable request.

## Abbreviations

BMI: Body mass index
CI: Confidence interval
NMW: The national minimum wage
MMSE: Mini-Mental State Examination
SABE: The Health, Well-being and Aging survey
