# Supplementary material for: Changes in prevalence of cognitive impairment and associated risk factors 2000–2015 in São Paulo, Brazil
Source: BMC Geriatr. 2021 Oct 27;21:609. doi: 10.1186/s12877-021-02542-x (PMC8554830; doi:10.1186/s12877-021-02542-x)
Supplement: Supplementary file 1 — Additional file 1: Table S1. Characteristics of the four waves (weighted estimates). [file 12877_2021_2542_MOESM1_ESM.docx]

**Supplementary Materials**

In Table S1, we display the characteristics of the respondents across the four waves with MMSE responses available.

Table S1. Characteristics of the four waves (weighted estimates).

| Characteristic  % (95% CI) | 2000 | 2006 | 2010 | 2015 |
| --- | --- | --- | --- | --- |
| Age-y |  |  |  |  |
| 60 - 64 | 33.7  (30.1-37.5) | 28.7  (20.5-38.4) | 32.2  (25.2-40.0) | 27.2  (20.8-34.8) |
| 65 - 69 | 27.3  (24.5-30.1) | 27.3  (23.0-31.8) | 22.1  (15.7-30.0) | 27.0  (22.0-32.6) |
| 70 - 74 | 18.9  (16.8-21.2) | 19.8  (16.5-23.5) | 17.7  (14.5-21.5) | 17.7  (12.3-24.8) |
| 75 - 79 | 11.2  (8.6-14.4) | 12.7  (10.5-15.2) | 12.8  (10.0-16.2) | 12.7  (9.7-16.6) |
| 80 - 84 | 5.8  (4.3-7.6) | 6.4  (4.8-8.4) | 8.2  (6.3-10.5) | 6.1  (4.4-8.3) |
| ≥ 85 | 3.3  (2.4-4.4) | 5.2  (3.6-7.3) | 7.1  (4.8-10.4) | 9.2  (6.8-12.4) |
| Mean age (*SD*) | 72.61 (8.07) | 74.27 (9.19) | 72.84 (9.59) | 71.44 (9.14) |
| Sex |  |  |  |  |
| Male | 41.2  (38.7-43.6) | 40.8  (37.9-43.7) | 40.0  (37.2-42.9) | 43.8  (41.2-46.4) |
| Female | 58.8  (56.4-61.3) | 59.2  (56.3-62.1) | 60.0  (57.1-62.8) | 56.2  (53.6-58.8) |
| Race^b^ |  |  |  |  |
| White | 70.6  (66.2-74.7 ) | 63.5  (58.9-67.9 ) | 58.7  (54.1-63.1 ) | 52.7  (48.8-56.6) |
| Mixed | 20.7  (17.1-24.8 ) | 20.0  (16.8-23.7 ) | 29.3  (25.0-34.0 ) | 37.4  (33.5-41.4 ) |
| Black | 4.0  (3.0-5.3 ) | 7.4  (5.7-9.5 ) | 6.7  (5.2-8.6 ) | 6.8  (5.1-9.0 ) |
| Other | 4.7  (3.3-6.5 ) | 9.1  (6.5-12.4 ) | 5.3  (3.8-7.3 ) | 3.1  (1.9-5.0 ) |
| Education^c^ |  |  |  |  |
| no education | 19.8  (16.4-23.8 ) | 15.4  (12.8-18.5 ) | 11.7  (9.6-14.2 ) | 8.5  (6.8-10.7 ) |
| primary | 65.5  (61.4-69.5 ) | 71.0  (67.2-74.5 ) | 66.7  (62.4-70.7 ) | 61.4  (57.7-65.0 ) |
| secondary | 8.8  (6.7-11.5 ) | 7.4  (5.6-9.7 ) | 12.8  (9.7-16.5 ) | 17.6  (15.3-20.2 ) |
| post-secondary | 5.8  (3.7-8.9 ) | 6.2  (4.1-9.3 ) | 8.9  (6.2-12.4 ) | 12.5  (9.5-16.3 ) |
| Mean education years (*SD*) | 3.75 (3.79) | 3.87 (3.79) | 4.81(4.21) | 5.50 (4.50) |
| Wages^c^ | |  |  |  |
| < 1 times NMW | 25.5  (22.5-28.8 ) | 25.9  (22.9-29.1 ) | 3.2  (2.2-4.5 ) | 4.4  (3.2-6.0 ) |
| 1-2 times NMW | 16.8  (14.5-19.5 ) | 26.5  (23.1-30.1 ) | 49.7  (45.1-54.3 ) | 49.3  (45.0-53.5 ) |
| 2-3 times NMW | 12.9  (11.0-15.1 ) | 16.3  (14.0-18.8 ) | 16.1  (13.8-18.6 ) | 18.1  (15.3-21.3 ) |
| 3-4 times NMW | 11.5  (9.8-13.5 ) | 10.9  (8.6-13.6 ) | 11.2  (9.1-13.7 ) | 15.5  (13.2-18.0 ) |
| > 4 times NMW | 33.3  (29.4-37.4 ) | 20.5  (17.1-24.4 ) | 19.8  (16.1-24.2 ) | 12.8  (10.3-15.9 ) |
| Cardiovascular risk factors | | |  |  |
| Stroke |  |  |  |  |
| no | 94.0  (92.6-95.2 ) | 92.1  (90.2-93.7 ) | 93.0  (91.4-94.2 ) | 93.1  (91.4-94.5 ) |
| yes | 6.0  (4.8-7.4 ) | 7.9  (6.73-9.8 ) | 7.0  (5.8-8.6 ) | 6.9  (5.5-8.6 ) |
| Diabetes^c^ |  |  |  |  |
| No | 81.5  (79.2-83.6 ) | 79.1  (76.4-81.6 ) | 74.9  (72.3-77.4 ) | 71.7  (68.8-74.5 ) |
| yes | 18.5  (16.4-20.8 ) | 20.9  (18.6-23.9 ) | 25.1  (22.6-27.7 ) | 28.3  (25.5-31.2 ) |
| Heart disease |  |  |  |  |
| No | 80.5  (78.4-82.4 ) | 77.9  (74.6-80.8 ) | 77.1  (74.3-79.7 ) | 76.2  (73.2-78.9 ) |
| Yes | 19.5  (17.6-21.6 ) | 22.1  (19.2-25.4 ) | 22.9  (20.3-25.7 ) | 23.8  (21.1-26.8 ) |
| Hypertension^d^ |  |  |  |  |
| No | 46.7  (43.7-49.6 ) | 37.4  (34.3-40.5 ) | 33.3  (30.4-36.3 ) | 33.7  (30.7-36.8 ) |
| yes | 53.3  (50.4-56.3 ) | 62.6  (59.5-65.7 ) | 66.7  (63.7-69.6 ) | 66.3  (63.2-69.3 ) |
| BMI^c^ |  |  |  |  |
| <18.5 | 2.8  (2.2-3.7 ) | 2.4  (1.6-3.4 ) | 1.3  (0.8-2.0 ) | 1.2  (0.7-2.0 ) |
| 18.5 - 24.9 | 35.3  (32.4-38.2 ) | 36.2  (32.9-39.6 ) | 25.5  (23.0-28.1 ) | 24.9  (22.2-27.9 ) |
| 25 - 29.9 | 39.8  (37.2-42.5 ) | 38.7  (36.2-41.2 ) | 41.0  (37.8-44.4 ) | 41.2  (37.9-44.6 ) |
| ≥ 30 | 22.1  (20.1-24.3 ) | 22.8  (19.8-26.1 ) | 32.2  (29.2-35.5 ) | 32.8  (29.0-36.8 ) |
| Depression |  |  |  |  |
| No depression | 81.0  (79.1-82.7 ) | 84.9  (82.3-87.2 ) | 80.9  (78.0-83.4 ) | 82.5  (79.9-84.9 ) |
| Mild depression | 15.5  (14.0-17.1 ) | 11.9  (10.0-14.2 ) | 15.9  (13.5-18.6 ) | 13.1  (11.0-15.6 ) |
| Severe Depression | 3.5  (2.7-4.6 ) | 3.2  (2.2-4.5 ) | 3.2  (2.2-4.6 ) | 4.4  (3.2-5.8 ) |

b *P* < .001 for difference between 2000 and 2015.

c *P* < .001 for difference between 2000 and 2010 and 2015.

d *P* < .001 for difference among 2000 and 2006- 2010 and 2015.
